# Time to change the way we think about tuberculosis infection prevention and control in health facilities: insights from recent research

**DOI:** 10.1017/ash.2023.192

**Published:** 2023-07-17

**Authors:** Tom A. Yates, Aaron S. Karat, Fiammetta Bozzani, Nicky McCreesh, Hayley MacGregor, Peter G. Beckwith, Indira Govender, Christopher J. Colvin, Karina Kielmann, Alison D. Grant

**Affiliations:** 1 Division of Infection and Immunity, Faculty of Medicine, University College London, London, UK; 2 TB Centre, London School of Hygiene & Tropical Medicine, London, UK; 3 The Institute for Global Health and Development, Queen Margaret University, Musselburgh, UK; 4 The Institute of Development Studies, University of Sussex, Brighton, UK; 5 Department of Medicine, University of Cape Town, Rondebosch, South Africa; 6 Africa Health Research Institute, Durban, KwaZulu-Natal, South Africa; 7 Division of Social and Behavioural Sciences, Faculty of Health Sciences, University of Cape Town, Cape Town, South Africa; 8 Institute of Tropical Medicine, Antwerp, Belgium; 9 School of Laboratory Medicine and Medical Sciences, College of Health Sciences, University of KwaZulu-Natal, Durban South Africa

## Abstract

In clinical settings where airborne pathogens, such as Mycobacterium tuberculosis, are prevalent, they constitute an important threat to health workers and people accessing healthcare. We report key insights from a 3-year project conducted in primary healthcare clinics in South Africa, alongside other recent tuberculosis infection prevention and control (TB-IPC) research. We discuss the fragmentation of TB-IPC policies and budgets; the characteristics of individuals attending clinics with prevalent pulmonary tuberculosis; clinic congestion and patient flow; clinic design and natural ventilation; and the facility-level determinants of the implementation (or not) of TB-IPC interventions. We present modeling studies that describe the contribution of M. tuberculosis transmission in clinics to the community tuberculosis burden and economic evaluations showing that TB-IPC interventions are highly cost-effective. We argue for a set of changes to TB-IPC, including better coordination of policymaking, clinic decongestion, changes to clinic design and building regulations, and budgeting for enablers to sustain implementation of TB-IPC interventions. Additional research is needed to find the most effective means of improving the implementation of TB-IPC interventions; to develop approaches to screening for prevalent pulmonary tuberculosis that do not rely on symptoms; and to identify groups of patients that can be seen in clinic less frequently.

Transmission of *Mycobacterium tuberculosis* (*Mtb*) within healthcare facilities presents a substantial risk to health workers and patients in communities experiencing a high burden of tuberculosis disease (TB).^1–6^

We recently concluded an interdisciplinary program of research with a focus on TB infection prevention and control (TB-IPC) in primary healthcare clinics in KwaZulu-Natal and Western Cape provinces, South Africa.^7,8^ These findings are discussed in light of recently published studies on TB-IPC, from other research teams, that also focus on primary healthcare clinics in countries with a high TB burden. Taken together, this evidence argues for a set of urgent changes to TB-IPC policy and practice. Some of these changes will also impact transmission of other airborne pathogens, eg, measles and SARS-CoV-2.

In our view, the key findings are applicable in similar settings in Southern and Eastern Africa with a high burden of HIV-associated TB, with some of the principles applicable more generally. We highlight in the manuscript where conclusions are based on modeling rather than empiric data.

## TB-IPC policy needs coordination and clear accountability

Interviews with stakeholders involved in policy debates in South Africa identified multiple barriers to the implementation of TB-IPC interventions.^9^ Despite TB being the leading cause of death in South Africa, advocates reported long-standing challenges in raising the level of concern about TB among donors, health departments, and even in local communities, leaving TB lower on the policymaking and policy implementation agenda than it should be.^9^ Institutional responsibility for TB-IPC is often fragmented across multiple roles, departments, budgets, and checklists, so it is unclear who is ultimately responsible for TB-IPC and accountable for preventing nosocomial transmission.^9^

TB-IPC necessarily involves multiple actors, including healthcare providers, those responsible for training health workers, and public works departments responsible for building and maintaining facilities. It is important, however, that budget lines for TB-IPC activities are clearly identified and that named individuals within government and the health service can be held accountable for implementation. Financial incentives for supporting TB-IPC need further thought. The South African health budget is structured by program, so ring-fenced budget lines for specific TB-IPC activities within each District Health Services program could be considered. As with IPC programs more broadly, most countries have policies in place, but far fewer invest adequately in supporting IPC leadership, implementation, and monitoring.^10–12^ Given *Mtb* transmission is difficult to measure programmatically, monitoring would need to focus on implementation of key TB-IPC interventions.

## Decongest clinics, reorganize patient flow

Modifiable direct determinants of *Mtb* transmission risk include the number of infectious and susceptible individuals present in a space; infectiousness, which is modified by TB treatment and by wearing a surgical mask; the use of personal protective equipment, such as N95 respirators; the ventilation rate; and the duration of exposure.^1^ Therefore, an obvious, and neglected, means of reducing *Mtb* transmission in clinics is to reduce the number of patients that concurrently occupy the same indoor space.^13^

In 11 facilities, we measured the amount of time people spent in clinic, where on the premises this time was spent, the occupancy of waiting areas, and how this changed over the measurement period.^13^ The median time spent in clinic was 2.5 hours with around 40% of attendees waiting for longer than 3 hours, the national target.^13^ Attendees spent most of their time in indoor spaces even where clinics did have capacity to seat patients in outdoor waiting areas.^13^ However, time indoors was reduced where the recommended path through the clinic included an outdoor waiting area.^13^ “Occupancy density” of indoor waiting areas was highly variable across locations and time. For example, in one clinic, smaller waiting areas that directly fed consultation rooms saw crowding in the afternoon, when the large main waiting area was relatively empty.^13^

Health system factors, including inadequate staffing levels in the public sector, are clearly a major determinant of clinic crowding.^14^ Addressing staffing levels is already a well-recognized public health priority.^15^ Building design can optimize patient flow—we discuss this later in the manuscript. On a smaller scale, regular low-intensity measurement of waiting times and patient flow may facilitate improvements in clinic performance and patient satisfaction, including identifying and responding to bottlenecks.^16^ Where possible, ensuring patients wait in well-ventilated spaces, ideally outdoors, will limit transmission risk. An existing evidence base^13^ supports community dispensing for chronic conditions and date–time appointment systems as interventions to reduce crowding in clinics. Practitioners seeking to implement these interventions should ensure sufficient administrative support and technological infrastructure.^17^

Reducing visit frequency might reduce crowding and, therefore, have positive secondary benefits in terms of TB-IPC. Studies, including a recent cluster-randomized trial of community-based HIV management,^18^ suggest it is safe to see HIV-positive people who are stable on treatment in clinic less frequently.^19^ Similar research on service delivery models for other chronic conditions would be valuable.

Reducing the frequency of clinic visits for some chronic conditions does not require sustained changes in practice by health workers already facing multiple other demands on their time. Indeed, such policies should reduce demands on clinic staff.

## Focus on undiagnosed pulmonary TB

In interviews we undertook with clinic staff in South Africa, risk of *Mtb* transmission was often perceived to be localized within spaces in which TB services were delivered.^20^ Less attention was paid to general waiting areas where transmission risk from undiagnosed pulmonary TB is more likely because infectiousness diminishes rapidly after starting effective TB treatment.^1^ This misperception of risk location has been noted by others, including access to TB-IPC training and respiratory personal protective equipment being preferentially offered to staff providing TB testing and treatment^21^; also administrative, domestic, and community health workers being denied access to these resources.^21^

We undertook a TB prevalence survey enrolling 2055 adults attending two primary healthcare clinics in rural KwaZulu-Natal.^22^ Most participants with *Mtb* cultured from sputum were attending routine appointments for HIV care.^22^ In keeping with this, Malawian data suggest that people who will be diagnosed as having TB in the subsequent 6 weeks are often present in clinics offering TB and HIV services, but that most of these individuals “did not give suspected TB as the reason for attendance.”^2^

TB prevalence was 1.0% (95% CI 0.6–1.5) in our clinic-based survey and 0.6% (95% CI 0.4–0.7%) in a community-based study conducted concurrently in the same area.^22^ Young men, a group known to be at higher risk of undiagnosed TB, were underrepresented in both surveys. That clinic prevalence was similar to community prevalence may be, at least in part, because less than half of primary healthcare attendances in South Africa are for acute illnesses.^13^ TB prevalence in attendees at primary healthcare clinics is notably different to that in inpatients in South African hospitals, where the prevalence of *Mtb* culture-positive sputum is around 20%.^3^

## Symptom-based administrative controls are probably insufficient

The first recommendation in the World Health Organization TB-IPC guidelines is to “triage … people with TB signs and symptoms, or with TB disease … to reduce *M. tuberculosis* transmission”; such administrative controls are argued to be the “first and most important component of any IPC strategy.”^23^ Symptom-based administrative controls are also central to the widely advocated FAST strategy.^24^

In our prevalence survey, we tested sputum regardless of patients’ symptoms. Among 20 participants with *Mtb* cultured from sputum, notably, 70% reported no TB symptoms (cough of any duration, night sweats, loss of weight, or fever) on the day of enrollment and would not have been identified through standard TB symptom screening.^22^

These results are consistent with evidence from community TB prevalence surveys in Asia and Africa in which 30–80% of individuals with *Mtb* culture-positive sputum had “subclinical” pulmonary TB.^25,26^ While this proportion may be overestimated, as some prevalence surveys used more restrictive symptom screening questions,^27^ it is clear that symptom-based criteria for TB testing will miss many adults with viable *Mtb* in their sputum.

To understand the implications for TB-IPC, we need to know the relative contribution that asymptomatic or pauci-symptomatic pulmonary TB make to *Mtb* transmission. Recent publications by epidemiologists and modelers have argued that asymptomatic individuals with pulmonary TB make a substantial contribution.^28–31^ There are limited data to support assumptions used in some of these analyses. However, the conclusions have biological plausibility, particularly where the bacillary load is high. In people with pulmonary TB, normal tidal breathing, rather than cough, could be responsible for most aerosolization of viable *Mtb*.^32–34^

While symptom-based administrative controls remain likely to reduce nosocomial transmission and the morbidity associated with late diagnosis, alternative approaches to screening are clearly needed.^35^ The results of the Targeted Universal Testing for Tuberculosis (TUTT) study, a cluster-randomized trial undertaken in 62 primary healthcare clinics in South Africa, suggest that a modest increase in TB diagnoses can be achieved by testing sputum from asymptomatic individuals with epidemiologic risk factors for TB disease.^36^ A recent meta-analysis suggests that point of care C-reactive protein (CRP) testing could play a role in screening for TB in HIV-positive outpatients.^37^ In outpatient settings with a high burden of both HIV and TB, the feasibility and cost-effectiveness of using these and other approaches to screen for TB disease as part of a TB-IPC intervention requires research.

## Proportionate regulation and co-design might make clinic buildings safer

Ventilation is a key determinant of *Mtb* transmission risk.^1^ There are few empirical estimates of the absolute ventilation rate in healthcare facilities in TB endemic settings.^38,39^ Many studies only report the “rebreathed fraction,”^40^ an approach that does not disaggregate transmission risk into that caused by crowding and that caused by poor ventilation. Using a combination of carbon dioxide (CO_2_) release experiments^41^ and paired indoor–outdoor CO_2_ measurements,^42^ we directly measured the absolute ventilation rate in 33 clinical spaces across 10 primary healthcare facilities.^43^ We found marked variation in natural ventilation between spaces, with the least-ventilated spaces being consultation rooms where doors and windows had been closed to allow air-conditioning units to work.^43^ Fully opening existing doors and windows led, on average, to a twofold increase in natural ventilation.^43^

We interviewed facility managers, government architects, and engineers about physical infrastructure in South African healthcare facilities (unpublished data). Several clinic managers reported that old clinic buildings were not “*fit for purpose*,” a legacy of historical inequalities in infrastructure provision. Building design can optimize ventilation and patient flow but, in the design of new facilities, architects and engineers must balance IPC concerns with other considerations, such as safety, temperature regulation, cost, and sustainability.

Changes in clinic populations and policies alter patient pathways and can unintentionally create new bottlenecks and overcrowding. Architects and engineers discussed the need for “*future proof*” building designs that would allow for greater flexibility in the use of space, including adaptation of patient pathways in response to changes in demographics, policies, or service provision. Architects and engineers also expressed a desire for more “*bottom up*” building design processes. This could incorporate co-design principles, involving consultations with health workers and colleagues monitoring service delivery.

Where new facilities cannot be built, both modeling and empirical data suggest that low-cost adaptations to existing buildings can markedly improve ventilation rates.^39,44,45^ However, facility managers reported that applications to adapt existing buildings were frequently hampered by cumbersome bureaucratic procedures, designed to prevent harm associated with poor-quality construction and financial mismanagement.^46^ Policymakers must urgently ensure that regulations better balance these concerns with the (likely more frequent) harms associated with nosocomial transmission of *Mtb* and other airborne pathogens. A more streamlined approvals process should be made available to individuals seeking to alter clinic buildings to protect patients and staff.

Our observation that thermal comfort places limits on the levels of natural ventilation that can be achieved has also been noted by others.^21,47^ South Africa experiences both hot summers and, in places, cold winters. We are currently using airflow modeling to investigate approaches to improving natural ventilation while maintaining thermal comfort. Upper-room ultraviolet germicidal irradiation (UVGI) may offer an alternative where extremes of temperature preclude sufficient natural ventilation.^47,48^ Properly installed UVGI is efficacious^49,50^ and safe.^51^ A history of poor installation and inadequate maintenance means UVGI is currently little used in South Africa. Work is ongoing to develop guidelines and delivery models to enable wider use.

## Implementation will not improve unless upstream, facility-level determinants are addressed

Upstream influences such as norms, values, relationships, power structures, and the policy environment—all potential drivers of poor implementation of IPC interventions—are neglected in the TB-IPC literature.^14,52^ In interviews with frontline staff, health system managers, patients, activists, researchers, and community members, we explored reasons TB-IPC interventions are often poorly implemented, despite general agreement that they are effective and fairly easy to employ.^20^

Nurses reported that masks created social and communication barriers between them and patients and heightened the stigma around TB.^20^ Both nurses and doctors described ways in which not wearing a mask was actively promoted by a local medical culture that privileged displays of invulnerability, strength, and individual responsibility.^20^ Many health workers perceived TB risk to be pervasive and thus normalized.^20^ Similar observations have been made in other settings.^21^

We used a participatory System Dynamics Modeling approach, to explore health systems constraints that prevent various TB-IPC interventions from being implemented.^53^ As part of the process, key stakeholders suggested changes that might overcome these constraints. These included strengthening supervision and monitoring of TB-IPC by operational managers and use of portable heaters to enable windows to be opened without compromising thermal comfort.^53^ Unless health systems constraints are considered and approaches to overcoming them included within planned interventions, TB-IPC interventions are likely to remain underutilized.^14,52^

## TB-IPC is highly cost-effective and needs investment

A mathematical model of *Mtb* transmission risk in eight primary healthcare clinics in KwaZulu-Natal and Western Cape suggests that relatively simple TB-IPC interventions could reduce the rate of transmission to clinic attendees by between 22% and 83%.^4^ Reorganizing care so that attendees wait in well-ventilated outdoor areas and installing upper room UVGI had the biggest impact in the model, but most interventions were predicted to be highly effective.^4^ This included interventions not typically considered part of TB-IPC, such as interventions designed to decongest clinic spaces.

Further modeling, using social contact data collected in the surrounding community, suggested that, in 2019, 4–14% of TB disease in the KwaZulu-Natal study community resulted from transmission in clinics, and that TB-IPC interventions in clinics could reduce community-wide TB incidence in 2021–2030 by 3–8%.^5^ A recently published molecular epidemiological study from Botswana concluded, similarly, that 3–8% of *Mtb* transmission resulting in TB disease might be attributable to contact in healthcare facilities.^6^

Cost-effectiveness analyses suggest that all of the TB-IPC interventions modeled, including combinations of these interventions, are highly cost-effective.^54^ We used the current opportunity cost-based threshold for South Africa, estimated at US$3,200 per disability-adjusted life-year (DALY) averted.^55^ The wearing of surgical masks by patients and N95 respirators by clinic staff, the intervention with the highest incremental cost-effectiveness ratio (ICER) in our model, costs only US$151 per DALY averted.^54^ The intervention with the highest impact compared to base case was the introduction of queue management systems with outdoor waiting areas (US$28 per DALY averted), followed by the installation, running, and maintenance of upper room UVGI (US$57 per DALY averted).^54^ Two interventions were estimated to be cost-saving compared to base case: optimizing the use of community dispensing for chronic conditions, which we modeled as an increase in the number of HIV-positive people with stable disease collecting their medications from external pickup points and modifications (retrofits) to clinic buildings to improve ventilation.^54^

These cost-effectiveness analyses included not only the costs of each intervention but also the costs of the necessary enablers to relieve constraints to intervention uptake and implementation, for example, additional targeted training and supervision at facility level.^53^ ICERs falling substantially below the cost-effectiveness threshold indicate that both the interventions and enablers are affordable and should be implemented.^54^

As discussed earlier, factors including stigma and comfort may preclude implementation of some TB-IPC interventions in specific clinics or at certain times of year. However, the wide range of cost-effective interventions available means feasible options should be available in most circumstances.

## COVID-19 and TB-IPC

IPC practice in South Africa changed during the SARS-CoV-2 pandemic. At the time of initially drafting this piece (July 2022), patients could not enter healthcare facilities without wearing surgical masks. Health workers wear surgical masks or N95/FFP3 respirators much more frequently. Social distancing is more common within healthcare facilities, and more facilities use outdoor covered waiting areas. Similar changes to health worker behavior with relevance for TB-IPC were seen in many other countries during the pandemic.

The TB-IPC research described above was largely undertaken before the pandemic. It remains unclear whether these positive changes will be sustained. While *Mtb* and SARS-CoV-2 differ in their modes of transmission, improvements in ventilation, outdoor waiting, and clinic decongestion, for example, would all be expected to reduce transmission of SARS-CoV-2 and other airborne pathogens.

## Conclusions

Much TB-IPC research and practice focuses narrowly on individual health worker knowledge of and (limited) adherence to a narrow set of interventions. We advocate reframing suboptimal TB-IPC as a health systems problem, rather than a problem caused by health workers not complying with guidelines.

In outpatient settings with a high burden of both TB and HIV, this would entail a set of specific changes. We suggest widening the range of interventions considered core to TB-IPC to include action to decongest clinics. Adequate physical infrastructure and administrative support are critical and require proportionate regulation, co-design, and planned flexibility to deal with changes in clinic use. Budget lines for TB-IPC interventions should be clearly identified, and it must be made explicit who is responsible for the implementation of policies. TB-IPC interventions are highly cost-effective, and health economic analyses support costing enablers into budgets to ensure they are delivered. Future research should focus on the relative effectiveness of different approaches to improving the implementation of TB-IPC interventions, identifying additional groups of patients that can be seen less frequently in clinic, and developing and evaluating symptom-agnostic approaches to screening clinic attendees for pulmonary TB.
